# Evaluating “Take the Stairs, Wyoming!” Through the RE-AIM Framework: Challenges and Opportunities

**DOI:** 10.3389/fpubh.2019.00368

**Published:** 2019-12-17

**Authors:** Laura E. Balis, Thomas Strayer

**Affiliations:** ^1^Division of Agriculture Cooperative Extension Service, University of Arkansas System, Little Rock, AR, United States; ^2^Center for Quality Aging, Vanderbilt University Medical Center, Nashville, TN, United States

**Keywords:** PSEs, RE-AIM, extension, evaluation, point-of-decision prompt, physical activity

## Abstract

**Introduction:** Health promotion delivery systems are increasingly being asked to implement policy, systems, and environmental interventions (PSEs). However, evaluating PSEs is challenging, especially in low-resource community settings. This paper describes the use of RE-AIM to evaluate a physical activity PSE delivered through University of Wyoming Extension and highlights challenges and opportunities in pragmatic, real-world program evaluation.

**Methods:** Extension health educators adapted a point-of-decision prompt intervention encouraging stairway use through posters, called Take the Stairs, Wyoming! *Reach* was assessed through estimates of daily traffic, *effectiveness* was assessed through opportunistic interviews, *adoption* was calculated as the number and proportion of sites that agreed to hang posters, *implementation* was calculated as the proportion of sites with a poster in place at a 2-weeks follow-up visit, and *maintenance* was assessed through 6-months opportunistic interviews (individual level) and proportion of sites with a poster in place (organizational level).

**Results:** Overall, the posters were widely *adopted* and most posters were *implemented* as intended. However, capturing *reach, effectiveness*, and *maintenance* was challenging, as health educators found the evaluation burdensome. Therefore, it was difficult to determine if the posters were effective at increasing physical activity levels.

**Discussion:** Suggestions are provided for capturing *reach, effectiveness*, and *maintenance* data in community settings. Future efforts are needed to create evaluation tools to pragmatically measure effectiveness of PSEs on changing behaviors, as well as to prioritize program evaluation in Extension.

## Introduction

Health promotion delivery systems are increasingly being asked to implement policy, systems, and environmental interventions (PSEs). PSEs, such as creating or improving places for physical activity (1) and providing healthier food and beverages in schools (2), focus on changing the environment to support healthy behaviors. One system tasked with implementing PSEs is the nationwide Land-Grant University Cooperative Extension System (Extension). In Extension, campus-based specialists support county-based educators who deliver programs in agriculture, natural resources, 4-H/youth development, community development, and family and consumer science (3). Within family and consumer science, Extension delivers health promotion programming addressing physical activity (since 2014) (4) and nutrition.

With its roots in home economics and agricultural education, Extension has a long history of implementing individual-level educational programs; however, implementing PSEs is a relatively new focus area. One driver of this change was the 2014 release of Cooperative Extension's National Framework for Health and Wellness, which outlined health promotion efforts based on the social-ecological model that included both “healthy and safe choices” and “healthy and safe environments” and identified PSEs as Extension priorities (5). Another factor is the Healthy, Hunger-Free Kids Act, which was released in 2010 and required the Supplemental Nutrition Assistance Program Education (SNAP-Ed, administered by Extension in some states) to implement comprehensive, multi-level interventions in addition to direct education (6). Lastly, funding opportunities available to Extension (e.g., Centers for Disease Control and Prevention grants) have shifted focus to increasing access to healthier foods and places for physical activity in an effort to create long-lasting health impacts (7).

Implementing PSEs in community settings has the potential for broad impacts on population health (1). However, evaluating PSEs can be challenging, as it is difficult to determine who is influenced by PSEs and track changes in their behavior. Evaluation of health promotion interventions (both PSEs and individual-level interventions) can be especially challenging in low-resource community settings (i.e., those that may not have funding or personnel dedicated to program evaluation) (8, 9). One challenge is that PSEs that were not designed and tested in community settings may include evaluations that are difficult to replicate (e.g., using many hours of observation pre- and post-intervention) (10). Adding to this challenge, interventions that are designed and evaluated in community settings as part of funded, researcher-initiated studies may also be difficult to replicate. Without funded research trials and dedicated evaluation staff, programs may not have the institutional support to be widely adopted and effectively evaluated, and consequently may not achieve the desired results (9).

Another challenge is that existing PSEs evaluation measures often only capture adoption and implementation at the organization level rather than measuring behavior change. For example, the PSEs listed in the SNAP-Ed Toolkit (a repository of practice-tested interventions used in SNAP-Ed) are primarily evaluated through indicators such as organizational-level adoption of nutrition or physical activity supports (11). The SNAP-Ed Evaluation Framework does include individual-level behavior change indicators; however, they are primarily designed to evaluate direct education (11).

While evaluating the impact of PSEs is difficult, it is necessary for stakeholder and funder accountability (12), as well as demonstrating the public value of federally funded programs, like Extension and SNAP-Ed (13). The reach, effectiveness, adoption, implementation, maintenance framework (RE-AIM) has been suggested for robustly evaluating PSEs (14), as well as for planning and evaluating Extension programs (15, 16). RE-AIM has been used for pragmatic program evaluation in community settings (8) and may help practitioners overcome the challenges to evaluating PSEs by providing a comprehensive evaluation framework. The purpose of this paper is to describe the use of RE-AIM as a planning and evaluation framework for a physical activity PSE delivered through University of Wyoming Extension (UWE).

## Methods

### Setting and Intervention

**Figure 1:**
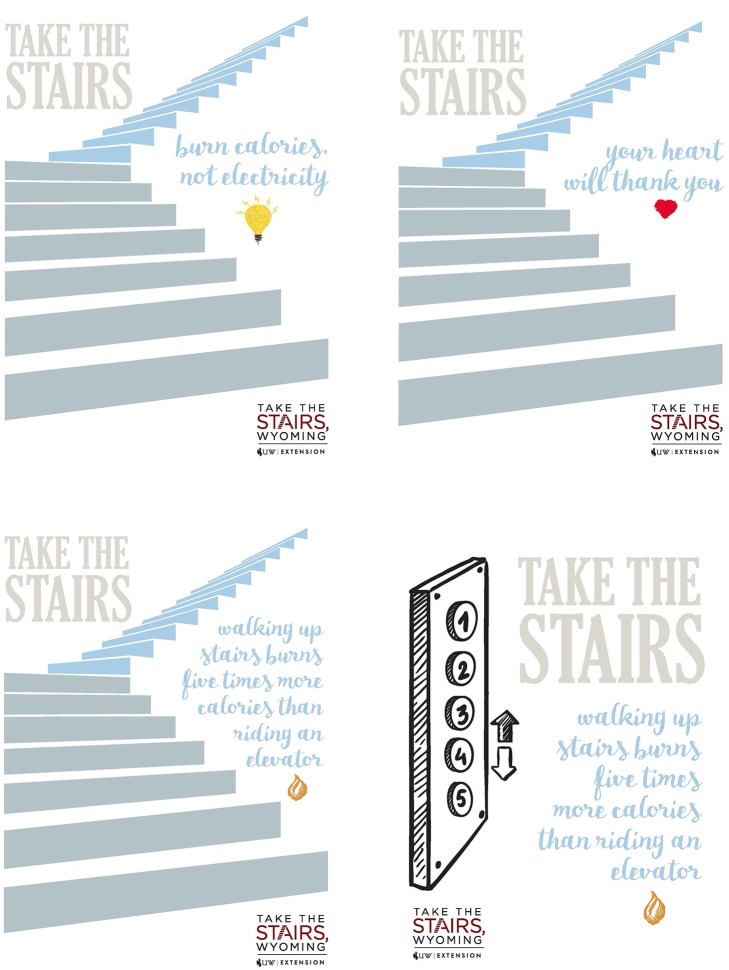
Take the Stairs, Wyoming! posters.

In Wyoming, five county-based Extension health educators deliver programs in three initiative areas: healthy eating, active living, and food safety; each educator covers multiple counties. Additionally, Cent$ible Nutrition Program (CNP) educators are located in most counties and are federally funded to serve limited-resource audiences through SNAP-Ed and the Expanded Food and Nutrition Education Program (EFNEP). The Extension health educators identified a need for an intervention to increase physical activity levels that was feasible to implement with a small number of Extension health educators covering the state. Collections of evidence-based interventions were searched, and point-of-decision prompts, recommended by the Community Guide (the Community Preventive Services Task Force's list of evidence-based strategies and interventions) (10, 17) were selected. The prompts encourage stairway use through posters to increase physical activity levels (18–20). The posters were adapted to give them a more modern look (see Figure 1). UWE program funds were used to print posters for statewide dissemination.

### Implementation Process and Research Design

The Extension health educators partnered with CNP educators to implement the intervention, titled Take the Stairs, Wyoming! As there was no database available listing all buildings with elevators in the state, each Extension health educator worked together with the CNP educator(s) in their area to identify businesses and organizations with elevators. Wyoming is a large, rural state with primarily small cities (95% of Wyoming cities have a population under 10,000); this made it possible for educators to identify buildings in their counties with elevators based on local knowledge.

Data were collected through an observational design. Both Extension health educators and CNP educators were asked to approach the identified businesses or organizations in their counties to hang the stairway posters and collect initial data (*reach* and *adoption*). After the initial visit, Extension health educators were responsible for completing data collection (*effectiveness, implementation*, and *maintenance*) through 2-weeks and 6-months follow-up visits. Educators were asked to implement the intervention between February and August 2018. The University of Wyoming Institutional Review Board approved this study.

### RE-AIM Measures

Detailed aims and outcome measures for each RE-AIM dimension are described below and summarized in Table 1. Means and standard deviations of continuous variables and frequencies and proportions of nominal variables were calculated in SPSS (IBM, Version 25).

**Table 1:** RE-AIM dimensions and measures.

**Dimension**	**Aims and Outcome Measures**
**Reach:** Number and proportion of individuals exposed to the PSE	Aim: To monitor and evaluate exposure rate Outcome Measure: Number of employees, building residents, clients, or daily traffic
**Effectiveness:** Impact on primary outcomes, quality of life, and unintended consequences	Aim: To confirm the effectiveness of the POD prompt posters at increasing stairway use Outcome Measure: Opportunistic interviews after 2 weeks
**Adoption:** Number, proportion, and representativeness of settings who deliver the intervention	Aim: To evaluate setting adoption rate Outcome Measure: Proportion and proportion of organizations/businesses that adopt the POD prompt posters
**Implementation:** Degree to which intervention was delivered as intended and the costs associated with continued delivery	Aim: To determine the degree to which POD prompt posters are delivered as intended Outcome Measure: Number of posters in place after 2 weeks
**Maintenance:** Long-term change in individual primary outcomes as well as extent to which delivery/ implementation is sustained over time	Aim: To determine the degree to which stairway use is sustained at least 6 months following intervention Outcome Measure: Opportunistic interviews after 6 months Aim: To determine the extent to which the posters are sustained after 6 months Outcome Measure: Number of posters in place after 6 months

### Reach

Each business or organization was asked to provide an estimate of daily traffic. For example, this could include average daily patrons at a library.

### Effectiveness

Extension health educators conducted opportunistic interviews (i.e., using a convenience sample of all individuals who walked past the poster) (21) for 1 h at each poster site at a 2-weeks follow up visit. The opportunistic interviews consisted of three questions: (1) “Did you see the poster?,” if yes, (2) “Did you feel that your behavior changed in response to the poster?,” and, if yes, (3) “How did the posters change your behavior?” This evaluation measure was selected after reviewing evaluation methods of all the literature that was included in the Community Guide recommendation and was selected as the most feasible. The other studies included in the Community Guide used up to 9 h of pre- and post-implementation observations, which was determined not feasible due to competing demands on Extension health educators' time.

### Adoption

Adoption was calculated as the number and proportion of businesses and organizations that agreed to hang the posters.

### Implementation

Implementation of the intervention (fidelity of posters implemented) was calculated as the proportion of sites that had a poster in place at the 2-weeks follow up visit to each poster site.

### Maintenance

Maintenance was assessed at a 6-months follow up visit to each poster site through opportunistic interviews (individual level) and the proportion of sites that had a poster in place (organizational level).

## Results

Eight Extension personnel approached businesses and organizations to place stairway posters across the state: three Extension health educators, four CNP educators, and one campus-based Specialist within the Department of Agriculture and Applied Economics who volunteered to assist. Posters were placed in eight of the state's 23 counties.

### Reach

At 38 of the 47 poster sites (81%), the estimated daily traffic was left blank or recorded as unknown, varies, not sure, or not available. Of the nine sites (19%) that did provide estimated daily traffic, an average of 99 (*SD* ± 127) individuals per site were reached.

### Effectiveness

**Figure 2:**
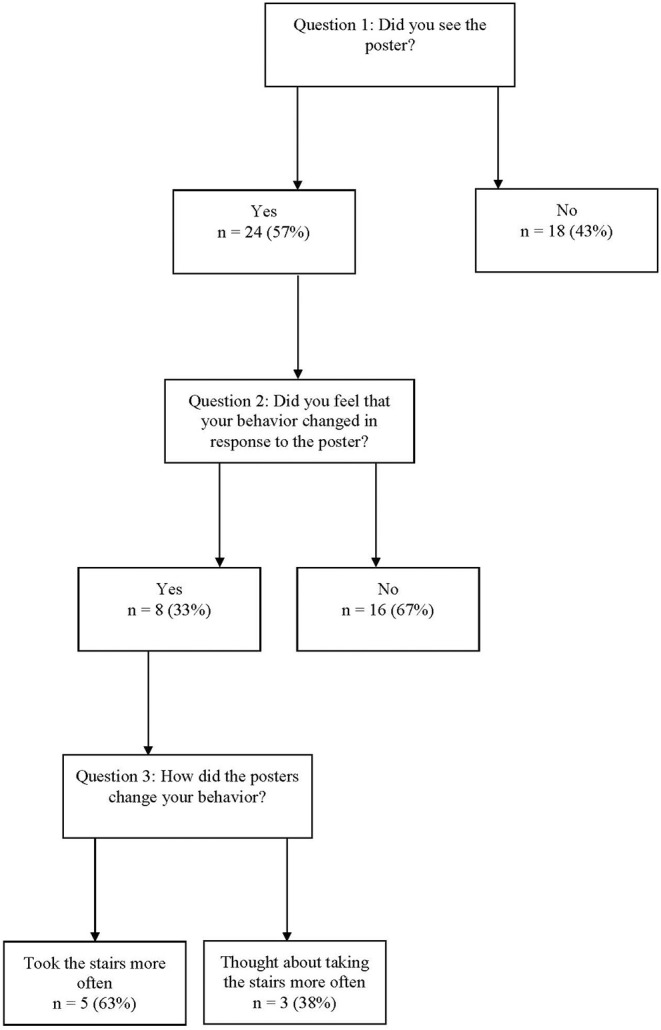
Results of opportunistic interviews (*N* = 42).

Opportunistic interviews were conducted at 10 poster sites (21%). Across these sites, 42 interviews were conducted. Twenty-four interviewees (57%) responded “yes” to question one indicating that they had seen the poster. Of these twenty-four, eight (33%) responded yes to question two indicating that they felt their behavior had changed in response to the poster. Of those eight, five (63%) responded that they had taken the stairs more often (e.g., “I came in with bags and would have taken the elevator, but saw the sign and took the stairs.”). Three (38%) indicated a change in their thoughts rather than their behavior (e.g., “It made me think twice about taking the elevator.”). Of the 16 who indicated they had seen the posters but their behavior had *not* changed, 11 provided unsolicited feedback indicating that they already take the stairs (e.g., “I always take the stairs.”) and three indicated that they had thought about changing their behavior (e.g., “I thought about it more seeing the poster.”). See Figure 2 for details.

These data were used to iteratively improve the intervention during the implementation phase (22). For example, when interviewees indicated that they had not seen the posters, this information provided an opportunity to place additional posters in locations that may have been more visible. This can be seen as a real-time adaptation to the intervention.

### Adoption

A total of 32 businesses and organizations were asked for approval to hang a poster. Of these, all but two (94%) provided approval, and a total of 44 posters were placed. During 2-weeks follow-up visits to these buildings, three additional posters were placed for a total of 47 posters.

### Implementation

Two-weeks follow-up visits were conducted at 42 of the original 44 poster sites; no additional follow-up visits were completed for the three additional posters placed during 2-weeks follow-up visits. Of the 42 follow-up visits completed, 29 posters (69%) were still in place. At two sites where the posters were no longer in place, staff reported the reason (e.g., the elevator was no longer working).

### Maintenance

As no follow-up visits were conducted, maintenance was not able to be reported.

## Discussion

Overall, the posters were widely adopted by the businesses and organizations that were approached and most posters were implemented as intended (i.e., still in place after 2 weeks). However, capturing reach, effectiveness, and maintenance was challenging. Taken together, these results suggest that the posters may not have been effective at increasing levels of physical activity through increased stair use, but it is difficult to determine due to the limited data. The challenges experienced with data collection as well as suggestions for improvement are presented.

### Data Collection Challenges

Determining *reach* was difficult, as most staff at the participating businesses and organizations were unsure of daily traffic. Additionally, proportion and representativeness (e.g., age, gender) were not captured, so it is unknown if the intervention reached those most in need. While a limitation for impact, this type of barrier is not uncommon in pragmatic settings, as this study actively worked in organizations focused on their own daily activities rather than those specifically recruited for research (23).

As for *effectiveness*, there were multiple issues with data collection. Three Extension health educators and the Specialist completed the 2-weeks follow-up visits, but only two of them completed the opportunistic interviews. Via email, one who did not complete interviews reported that it was too time consuming and not a good use of her time. Of the two staff members who did complete opportunistic interviews, at four sites they were not completed as they were not able to obtain permission from staff at the business or organization. Additionally, at six of the sites where interviews were conducted, the interview period lasted for less than the prescribed 1 h; one Extension health educator reported that this was due to time constraints when traveling to distant sites. Of the 42 interviews that were completed, only five interviewees reported an actual change in behavior as a result of seeing the posters. Additionally, as the majority of interviewees who had seen the posters indicated that they already take the stairs, the targeted population may not have been reached through the poster intervention. In the future, a follow-up question for those who saw the poster but did not change their behavior may be useful to provide insight into improving effectiveness.

The issues faced in collecting effectiveness data also made collecting *maintenance* data challenging; if staff experience difficulty with data collection methods at the start of the intervention, it is likely that they will continue to struggle with completing evaluations 6 months or more post-program. No staff completed the prescribed 6-months follow-up visits, so no maintenance data were able to be reported.

### Challenges in Evaluation

The barriers experienced in evaluating this PSE—especially effectiveness and maintenance data—are common among community organizations, as they often do not have the means to monitor impacts of PSEs on behavior change (14). Overall, more work is needed to evaluate PSEs in low-resource, community settings. Organizational changes, along with more feasible measures, could improve PSE evaluation in the future.

#### Need for Organizational Changes

One of the main barriers in this study was the lack of adherence to data collection by Extension health educators. Indeed, Extension struggles with program evaluation; collecting empirical data on behavior change as a program outcome is still relatively novel to the system (8, 9, 24, 25). In the case of PSEs, which are also fairly new to Extension and more difficult to evaluate than direct education interventions, matching evaluation methods to staff resources and expectations is key (Balis et al., under review). While this intervention was selected and planned by a fellow Extension health educator through a participatory approach (26–28), the evaluation was still considered a burden. This perception of evaluation as onerous highlights the need to change Extension culture to prioritize time spent evaluating programs rather than only time spent delivering programs. However, part of this burden must still remain on intervention developers to continuously consider the feasibility of the intervention's outcome measures.

#### Need for Feasible Measures

To improve data collection adherence, feasible measures that are less of a burden on staff need to be available. Intervention developers should consider including pragmatic, low-cost evaluation measures with their interventions for community organization staff to select. For example, with additional funding, infrared people counters or open/close sensors on doors throughout adoption organizations are relatively low-cost solutions that could be used to collect pre- and post-intervention data. These types of measures reduce staff time while providing an estimate of people using stairs and also estimate (if placed at multiple levels) how many flights of stairs individuals will use. Additionally, they would provide an objective measure of physical activity rather than the subjective measure used in this study. These feasible, objective measures need to be tested and, if successful, included in program repositories (e.g., the SNAP-Ed toolkit and evaluation framework) (11) to be used by professionals in community-based organizations. Finally, engaging in partnerships may also reduce evaluation burden. For example, students could complete observations or interviews for research experience; however, this can present an obstacle for Extension interventions that are located throughout the state rather than clustered near campus. Partnering with the organizations and businesses that adopt stairway posters and training their staff to collect effectiveness and maintenance data (e.g., through systematic observations) could also result in better data completion (14). The intervention may have been improved by engaging these stakeholders during the planning process.

There were some limitations to this study, including small sample sizes and incomplete data collection. However, we believe that it is important to include these data in an effort to highlight the reality of real-world program implementation and evaluation. There have been calls from organizations such as the National Institute of Aging (29), funding announcements from the National Institute of Health (30), and commentary pieces from the New England Journal of Medicine (31) that all discuss the various important reasons for conducting pragmatic research. To summarize these points, the real world does not conform to the unrealistic expectations of a randomized-control trial, and while these trials are incredibly important during efficacy testing, it is equally important that intervention are adaptable to real-world uncontrolled settings. The barriers within this study highlight these pragmatic needs.

Overall, RE-AIM was a useful tool for both planning and evaluating this intervention; as recommended, it can also be used after delivery to iteratively refine the intervention (22). For example, for the next iteration, the needs of Extension health educators who did not adhere to data collection procedures can be considered to tailor the evaluation plan to better meet their needs or provide training and technical assistance. Additionally, future iterations could be adapted through RE-AIM to reach businesses and organizations with populations that do not already take the stairs (e.g., through engaging the organizations to complete pre-intervention observations).

Implications for intervention developers include providing PSE evaluation tools that go beyond assessing adoption and implementation and are feasible to use in low-resource community settings. Using pragmatic measures (32) could allow community organizations to confirm effectiveness of PSEs while also collecting data on the other RE-AIM dimensions to ensure these interventions work in the “real world.”

## Data Availability Statement

The dataset analyzed during the current study is available from the corresponding author on reasonable request.

## Ethics Statement

This study involving human participants was reviewed and approved by University of Wyoming IRB. Written informed consent for participation was not required for this study in accordance with the national legislation and the institutional requirements.

## Author Contributions

LB conceived of the study, participated in its design and coordination, and led the manuscript preparation. TS contributed to data analysis and manuscript preparation. All authors read, contributed to, and approved the final manuscript.

### Conflict of Interest

The authors declare that the research was conducted in the absence of any commercial or financial relationships that could be construed as a potential conflict of interest.
